# Operationalizing a real-time scoring model to predict fall risk among older adults in the emergency department

**DOI:** 10.3389/fdgth.2022.958663

**Published:** 2022-10-31

**Authors:** Collin J. Engstrom, Sabrina Adelaine, Frank Liao, Gwen Costa Jacobsohn, Brian W. Patterson

**Affiliations:** ^1^ Department of Emergency Medicine, UW-Madison, Madison, WI, United States; ^2^ Department of Computer Science, Winona State University, Rochester, MN, United States; ^3^ Department of Enterprise Analytics, UW Health, Madison, WI, United States; ^4^ Department of Biostatistics and Medical Informatics, UW-Madison, Madison, WI, United States

**Keywords:** falls prevention, EHR, risk stratification, machine learning, AI, precision medicine

## Abstract

Predictive models are increasingly being developed and implemented to improve patient care across a variety of clinical scenarios. While a body of literature exists on the development of models using existing data, less focus has been placed on practical operationalization of these models for deployment in real-time production environments. This case-study describes challenges and barriers identified and overcome in such an operationalization for a model aimed at predicting risk of outpatient falls after Emergency Department (ED) visits among older adults. Based on our experience, we provide general principles for translating an EHR-based predictive model from research and reporting environments into real-time operation.

## Introduction

Predictive models have the potential to transform clinical care by providing clinical decision support, but only when implemented correctly. A large body of literature exists on the development of models using existing data (1–6), and an increasing number of studies have additionally focused on the importance of designing appropriate interfaces to present the output of models to clinicians (7–9). Less focus has been placed on the technical and system challenges of operationalizing these models by running them in clinical environments in which they can function in real time. This case-study describes challenges and barriers we overcame in the use of such a model after it had been created and validated in silico. Based on this experience, we provide general principles for translating an EHR-based predictive model from research and reporting environments into real-time operation.

### Case: Preventing falls after ED visits

Falls are the leading traumatic cause of both injury and death among older adults (age ≥ 65 years) (10). Over 3 million patients who have fallen and require medical care present to US emergency departments (EDs) every year (11); however, the ED itself has not traditionally played a major role in outpatient fall prevention (12). In our health system, 34% of patients presenting to the ED for a fall have had at least one ED visit in the prior six months (13), demonstrating a missed opportunity to connect patients with existing clinical interventions to reduce future fall risk.

Our research team has developed and validated an innovative automated screening algorithm that uses machine learning coupled with electronic health record (EHR) data to predict fall risk in the 180 days following an ED visit using retrospective data (14). This algorithm had the promise of identifying older adults at high risk of falling in the 6 months following the ED visit. Furthermore, engaging with experts in human factors engineering and clinicians, the study team designed a workflow and alerts designed to create a system in which the algorithm facilitates screening of older adult patients in the ED and facilitating referral for fall prevention services (15). Fulfilling this promise required successful translation of the predictive screening algorithm to hospital IT systems and clinical care.

Our task was to operationalize a functional model derived from a research dataset into production. Real impact depended on the ability to translate the research model into a corresponding operational model with minimal effects on model performance.

## Steps to operationalization

To be successful, we needed to overcome the translational barriers involved in implementing a real-time machine learning model for predicting older adult ED patients at highest risk for a fall event during the following 6 months. In early meetings between the operational and research teams, we identified several issues with the research model which necessitated changes before implementation would be possible. Firstly, some features used in the research model, while theoretically referring to events that happened in the ED, would not be accessible for use in real time during ED visits. This empirical issue is sometimes referred to as “data latency” or “time travel”, where the retrospective data set does not appropriately reflect the real-time availability of the features (16, 17). In our case, diagnosis codes referring to the ED visit were added not only by clinicians at the time of the visit but by professional coders several days later. Additionally, based on our data infrastructure, there was a computational and maintenance advantage to simplifying our model type and decreasing the number of features. For this reason, the diagnosis codes were left out of the final machine learning model during the operationalization phase.

**Figure 1:**
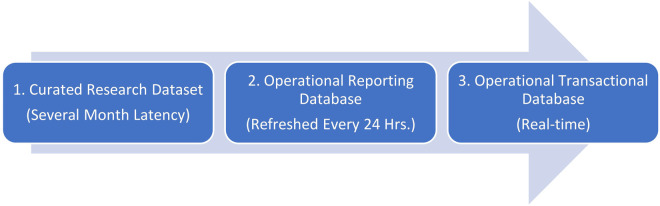


Our model was implemented in three stages. Time from initial discussion with operational stakeholders to active deployment to clinic front line staff was a total of 15 months. As shown in Figure 1, the overall process can be thought of as three stages, ranging from training and testing on a research dataset in *Stage 1* to a production-side validation in *Stage 2* to a live implementation in *Stage 3*.

### Stage 1: Research dataset

The research dataset used for training and testing consisted of 9,687 instances from patient visits to the Emergency Department (ED) over a span of three and a half years. Roughly 725 features relating to vital signs, past diagnoses, and demographics were selected for the modeling process. In the end, six models were chosen based on area under the ROC curve (AUROC). The AUROC performance of these models ranged from 0.72 for logistic regression to 0.78 for forest-based prediction algorithms (18). Prior to moving from the research environment, we trained models using fewer features and were able to maintain performance while paring down to 15 features. Features involving historic diagnosis data, vital signs, and lab values were ultimately left out, as they were not as predictive as initially thought. Height, weight, and age were found to be strong predictors of future falls and were retained, along with those features pertaining to patients' mobility assistance, dementia status, and past occurrences of at-home falls.

### Validation in reporting database

Validating our model on the production side during the second stage involved collaboration with the health system's applied data science team. Before moving the model into a real-time scenario, we first validated it using our operational reporting database. This database, while theoretically containing the same information as the research database, required re-querying for features used in our research model to match reporting needs for the production model which would gather data from the electronic health record. This was accomplished by issuing SQL queries to the database one-by-one for features of interest. These features were then fed into the models developed in *Stage 1* and evaluated on the same metrics. This process resulted in an AUROC of 0.69 for a production-ready logistic regression model, a slightly lower, but still acceptable, performance for selecting the most at-risk patients.

### Implementation in production transactional database

Planning for final model deployment involved a partnership between physicians, data scientists, computer scientists, health services researchers, and industrial engineers. Ultimately, the features validated in Stage 2 were retrieved from the operational transactional database and forwarded to a model deployed in the cloud, which returns a patient risk score to the EHR. In a separate publication we describe the design of the physician facing interface, an interruptive alert which fires when the returned risk score is above a threshold value. This alert notifies ED clinicians of patients' elevated risk of future fall and facilitates a referral order for our outpatient fall prevention services after an ED visit (15).

## Challenges

In moving from *Stage 1* to *Stages 2* and *3*, several unforeseen **feature translation considerations** presented themselves. One of the conveniences of *Stage 1* was the availability of a curated research dataset generated from patient visits. The features from this dataset had been cleaned, however, with some features being removed and new features being added that were derived from others in the dataset. Mapping features to the operational dataset necessitated re-querying features directly from the same data source that would be used in production. Maintenance of the model would require evaluating the consistency of features over time in production data, which would be challenging with so many features. For this reason, the feature set was pared down to include a final production of 15 features. Additionally, ICD data used in the research dataset was not available in real time. For this reason, when moved to the real-time environment we substituted chief complaint data for the ICD data (19).

After these feature-related challenges were overcome, our model was able to compute a risk score for each patient based on the 15 features, all available at time of visit. In our *Stage 1* research, random forest-based models outperformed every other model; however, the difference in practice between these and regression models was minimal. **IT constraints** existed to operationalizing a random forest-based model; as this was the first such model being put into production, there was a strong operational preference for a regression-based model for simplicity of implementation.

From a provider standpoint, this change made sense as well. Providers tend to trust more transparent models that are more explainable (20). Logistic regression is comprised of a linear combination of variables, the importance of which is determined by coefficients that can be interpreted by providers and compared to what they know about falls risk. A desire to ensure we had an **interpretable model** further influenced our choice to pursue regression rather than tree-based models. Also noteworthy was that the physicians interpreting model performance were interested in number needed to treat (NNT) at a given operational threshold (14), a clinical measure that summarizes interventional effectiveness by estimating the number of patients referred to the clinic to prevent a single future fall, rather than AUROC. In summary, our model started as a 700+ feature random forest in the research space but was adapted to a 15-feature regression model for our first operational deployment. This resulted in a small decrease in AUROC and small increase in NNT; however, given the advantages in ease of deployment and maintenance, this was seen as an acceptable tradeoff.

In our research phase, six models (i.e., standard linear and logistic regression, ridge logistic regression, LASSO logistic regression, AdaBoost, and random forests) were tested. For simplicity, the logistic regression model was ultimately chosen as the only one used in production to predict the likelihood of falling six months after leaving the ED. After choosing a model, the **threshold** at which it fired needed to be specified. The clinic to which the intervention referred patients had constraints on the number of patients that they could accommodate each week. This required the model threshold to be adjusted to flag a number of patients commensurate with the operational referral capacity. Our ability to describe thresholds based on both the number of likely referred patients and the NNT among this group allowed all stakeholders to understand the implications of threshold selection and ongoing adjustment. We have developed a free toolkit which allows calculation of projected NNT at various model thresholds for predictive models, available at www.hipxchange.org/NNT.

Finally, as part of implementing the model in the electronic health record, a point for **model placement in the workflow** had to be chosen so that an interruptive alert would fire, informing the provider to the patient's fall risk. Since all features were available at the time of discharge, this was chosen as the time for the model to run. We describe the design of the alert interface separately (15), but note here that ideal workflow placement of the alert was not achievable, as we were forced to fire the alert at a time when all necessary information to assess patient eligibility was already electronically available in the chart, and further in an area of the chart which was a required portion of the workflow for all discharged patients, to ensure providers would see the alert.

## Discussion

### Key considerations and questions

While there is increasing recognition that implementation of predictive models requires appropriate validation and governance, the act of moving models from a research platform to operational use presents a unique set of more mundane challenges. In addressing the issues as they arose during the deployment of an EHR-based fall risk prediction model, we identified a series of questions which needed to be addressed. We group these questions below into five domains, summarized in Table 1 along with examples of our own adaptations in response to these considerations. In future projects, we have found this set of considerations to provide a useful checklist for operationalization.

**Table 1:** Guiding considerations: from research to practice.

Consideration for Implementation	Research Design	Operational Adaption
Translational Considerations	725 Features	15 Features
IT Constraints	Tree-Based Models	Logistic Regression
Model Interpretation	Tree-Based Models	Logistic Regression
Communicating model performance and thresholding	Area Under ROC Curve (AUROC) and various NNT thresholds	Adjustable threshold chosen based on NNT and operational capacity
Model Placement in Workflow	Not Considered	Discharge Navigator in Emergency Department

#### Feature translation considerations

Do the features that were used to develop the model exist in the context in which the model will be receiving data? Are any features no longer available, or do they change between real-time and retrospective queries? Does the gain in performance from additional features justify the effort needed to create and maintain a more complex model? In our case, review of features from our model revealed that some were not available in real-time. In particular, a diagnosis was thought to be entered only by a physician in the research phase, but most diagnoses are actually entered after a patient visit by professional coders. For this reason, the “diagnosis” feature from the research model was excluded from the production model. Among the features that were available, many did not add enough to our model to justify the additional maintenance and complexity of including them. For example, vital signs and many historical diagnoses were features that were part of the research set but were ultimately left out of the production model for lack of predictive value.

#### IT constraints

Is it possible for the organization to implement the model? How might model choice be influenced by a healthcare institution's EHR hardware and software? How can models be kept as simple as possible during implementation? In our case there was a preference for a simpler regression-based model for our first attempt at real time prediction to simplify our technical workload, since this time we have iteratively built to more complex model types for other use cases.

#### Model interpretation

How will model choice impact provider trust? What metrics will such providers use in assessing model viability? In our case, an additional consideration for moving from tree-based to regression models was the ease of communicating model features and operations to our clinical staff.

#### Communicating model performance and thresholding

What cut-off should be chosen for a model in classifying patients? How should it be chosen based on model performance and clinical scenario? For our clinical scenario, an adjustable threshold based on projected number of patients referred per week by the model, with the resultant performance expressed in NNT, proved a valuable asset in gaining model trust from our referral partners.

#### Model placement in workflow

When in the user's workflow is sufficient data to run the model entered into the EHR? How quickly will a score need to be calculated in order to be displayed back to the end user in time for action? Is there a distinct electronic trigger that can be used to act on a model score? In our case, responding to these technical considerations significantly impacted our clinical decision support (CDS) design process; in order to collect the required inputs before sending an alert, we were forced to place the tool later in the workflow than our design indicated was optimal.

## Conclusion

As machine learning has seen wider uptake in the healthcare setting, there has been an increased need for translating models developed in silico to the bedside. Our team successfully migrated one such model focused on at-home falls risk to our university's emergency department. The process of doing so revealed several challenges which do not fall explicitly within the realm of model development and validation or within the traditional scope of intervention design from a physician workflow perspective.

Ultimately, these challenges were surmountable, but our experience suggests that model operationalization should not be considered a purely technical barrier to implementation but given early consideration when planning an intervention. We hope that the considerations presented here provide guidance for future translation of models into “the wild” and, more generally, bridge the gap that currently exists between research and practice where modeling techniques are concerned.

## Data Availability

The datasets presented in this article are not readily available because **models were created using potentially identifiable patient data used for quality improvement. Due to patient privacy concerns, we are unable to make this data public**. Requests to access the datasets should be directed to **Brian Patterson,** **bpatter@medicine.wisc.edu**.
